# Circular RNA, circular RARS, promotes aerobic glycolysis of non‐small‐cell lung cancer by binding with LDHA

**DOI:** 10.1111/1759-7714.14758

**Published:** 2023-01-11

**Authors:** Haoran Li, Qi Huang, Haifa Guo, Xiuyuan Chen, Xiao Li, Mantang Qiu

**Affiliations:** ^1^ Department of Thoracic Surgery Peking University People's Hospital Beijing China; ^2^ Thoracic Oncology Institute Peking University People's Hospital Beijing China; ^3^ Department of Thoracic Surgery The First Affiliated Hospital of Zhengzhou University Zhengzhou China; ^4^ The First Department of Thoracic Surgery, Beijing Chest Hospital Capital Medical University Beijing China

**Keywords:** aerobic glycolysis, circRARS, LDHA, NSCLC, prognosis

## Abstract

**Purpose:**

Accumulating evidence has highlighted the critical roles of circular RNAs (circRNAs) in non‐small‐cell lung cancer (NSCLC). This study aims to unveil the roles of circRARS (circular RARS) (hsa_circ_0001551) in NSCLC.

**Methods:**

Quantitative real‐time PCR was used to determine the expression of circRARS in NSCLC tissues and cells. Kaplan–Meier analysis was used to determine the prognostic value of circRARS expression. CCK8, transwell, and wound healing assays were used to assess the proliferation, invasion, and migration abilities of NSCLC cells. RNA pull‐down, cell fraction, glucose consumption, lactate production, and lactate dehydrogenase activity assays were conducted to explore the potential mechanisms of circRARS in NSCLC.

**Results:**

circRARS is upregulated in NSCLC tissues and positively correlated with smoking status, lymph node metastasis, and higher tumor stages. NSCLC patients with high expression of circRARS have poor overall survival. Functional assays demonstrated that circRARS accelerated the proliferation, invasion, and migration of NSCLC cells in vitro. The cell fraction suggested that circRARS mainly accumulated in cytoplasm and the RNA pull‐down assay showed lactate dehydrogenase (LDHA) could bind with circRARS. Furthermore, circRARS positively regulates LDHA activity and LDHA expression at the transcription level. Moreover, downregulated circRARS decreases glucose consumption and lactate production and compromises aerobic glycolysis in NSCLC cells. Finally, rescue assays showed circRARS could promote NSCLC cell proliferation by regulating LDHA activity.

**Conclusion:**

This study shows that circRARS can promote glycolysis and tumor progression in NSCLC by regulating LDHA.

## Graphical abstract

circRARS (circular RARS) is upregulated in non‐small‐cell lung cancer (NSCLC) tissues and positively correlated with smoking state, lymph node metastasis, and higher tumor stages. NSCLC patients with high expression of circRARS have poor overall survival. Moreover, circRARS accelerates the proliferation, invasion, and migration of NSCLC cells in vitro. Furthermore, circRARS positively regulates lactate dehydrogenase LDHA activity and its expression in transcription level. Downregulated circRARS decreases glucose consumption and lactate production, and compromises aerobic glycolysis in NSCLC cells. In short, circRARS can promote glycolysis and tumor progression in NSCLC by regulating LDHA.

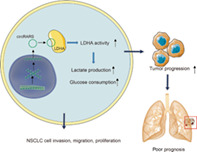

## INTRODUCTION

The incidence of lung cancer (LC) has been increasing rapidly, and the morbidity and mortality rates were both the first in China.^1^ Histologically, non‐small‐cell lung cancer (NSCLC) accounts for approximately 85% of LC cases and the 5‐year overall survival (OS) rate remains very poor.^2^ Although LC has yielded encouraging results with early screening in high‐risk populations of NSCLC and substantial progress has been made in targeted therapy and immunotherapy, the OS has not improved significantly.^3^, ^4^ It is therefore urgent to promote our understanding of NSCLC pathogenesis and continue to seek new targets for the occurrence and progression of diagnostic and therapeutic strategies.

Circular RNAs (circRNAs), produced from “back‐splicing” of primary transcripts, are covalently closed and endogenous biomolecules with tissue‐specific, cell‐specific, and development‐specific expression patterns.^5^, ^6^ circRNAs exert regulatory functions in various biological functions as miRNA or RNA binding protein (RBP) sponge and encode some functional peptides in human cancers.^7^ Moreover, the aberrant expression of circRNAs commonly exists in tumorigenesis and the development of LC, playing oncogenic or antioncogenic roles and affecting cellular functions such as proliferation or migration.^8^ For instance, circPRKCI binding to miR‐545 and miR‐589 promotes the protein expression of the transcription Factor E2F7 in lung adenocarcinoma progression.^9^ Although several NSCLC‐related circRNAs have been identified,^10^, ^11^, ^12^, ^13^, ^14^ the mechanisms by which circRNAs promote the progression of NSCLC still need to be further elucidated.

Aerobic glycolysis, also called the Warburg phenomenon, is the energy metabolism characteristic of tumor cells and results in the malignant phenotype of tumor cells, exhibiting an increasing rate of glucose consumption and lactic acid production.^15^, ^16^, ^17^ Lactate dehydrogenase (LDHA) is an enzyme that catalyzes the conversion of NADH and pyruvate to NAD+ and lactate to regulate glycolysis.^18^ LDHA knockdown reduced glycolysis and compromised proliferation in cancer cells and in mouse models.^19^ Notably, several circRNAs have been reported to regulate LDHA through different mechanisms in different cancers such as at the activity^20^ and transcription levels.^21^

Herein, this study was performed to uncover the roles of circRARS (circular RARS) (hsa_circ_0001551) in NSCLC progression and glycolysis. We found that circRARS can promote NSCLC development and that higher expression of circRARS was closely correlated with an unfavorable prognosis of NSCLC patients. Further investigation indicated that circRARS can bind to LDHA and promote aerobic glycolysis. In summary, our results demonstrated the critical roles of circRARS in NSCLC, which may provide a promising therapeutic target.

## MATERIALS AND METHODS

### Human tissue samples and cell lines

A total of 90 pairs of NSCLC tissues and paired adjacent normal tissues were collected from Peking University People's Hospital (Beijing, China). All patients received lobectomy or wedge resection without any preoperative radiation or chemotherapy. The clinical data and follow‐up information were also collected. This study was approved by the Ethics Committee of Peking University People's Hospital. Each patient included in this study provided written informed consent.

NSCLC cell lines (A549, SPC‐A1, NCI‐H1703, NCI‐H1299, and H1975) and 16‐HBE cells were purchased from the Cell Bank of the Chinese Academy of Sciences (Shanghai, China). SPC‐A1 and 16‐HBE cells were cultured in Dulbecco's modified Eagle's medium (DMEM; Gibco) and others were cultured in RPMI 1640 medium (Gibco). The cell lines were identified by short tandem repeat (STR) and tested for mycoplasma contamination. All media were supplemented with 10% fetal bovine serum (FBS) and 1% penicillin and streptomycin. All cell lines were cultured at 37°C with 5% CO_2_ in a humidified incubator.

### RNA isolation and quantitative real‐time PCR

Total RNA was extracted from NSCLC tissue samples and cells using TRIzol reagent (Invitrogen). For the RNase assay, 5 μg of RNA from cells was incubated with or without RNase R (Epicenter Technologies) for 15 min at 37°C. RNA isolation of nuclear and cytoplasmic fractions of circRARS was assessed by a PARIS Kit according to the manufacturer's protocol (Ambion). GAPDH and snRUN1 served as cytoplasmic and nuclear markers, respectively. Reverse transcription (RT) was performed according to the manufacturer's protocol (Takara). For qRT‐PCR, each quantitative polymerase chain reaction was performed in an Applied Biosystem with a total reaction volume of 10 μl (Takara). GAPDH was used as an internal control and all experiments were performed in triplicate. The primers used in this study are showed in Table S1.

### Genomic DNA and nucleic acid electrophoresis

Genomic DNA (gDNA) was extracted from NSCLC cells according to the protocol of the PureLink Genomic DNA Mini Kit (Thermo Fisher Scientific). The complementary DNA (cDNA) and gDNA were analyzed by 2% agarose gel electrophoresis with 1% Tris Acetate‐EDTA buffer (TAE) buffer at 120 V for approximately 30 min. The bands were detected by UV irradiation.

### siRNA and plasmid construction

circRARS was silenced by small interfering RNAs (siRNAs) targeting the back‐splice junction of circRARS. The siRNAs were synthesized by Tsingke Biotechnology. Full‐length circRARS cDNA was synthesized by Tsingke Biotechnology and cloned into the pCDNA3.1 vector. NSCLC cells were seeded in six‐well plates over 24 h and were transfected with specific siRNA, control siRNA (NC), and plasmids using Lipofectamine 3000 according to the kit instructions (Thermo Fisher Scientific). The efficacy of silencing or overexpression was assessed after 24 h of transfection via RT‐PCR.

### Cell proliferation, invasion, and migration

Cell proliferation was evaluated by CCK‐8 assay (Biosharp) according to the manufacturer's protocol. Briefly, cells were seeded in 96‐well plates with 100 μl of medium and 10 μl of CCK‐8. After incubation for 2 h at 37°C, the absorbance of each well was measured with a microplate reader at 450 nm. Transwell assays with Matrigel were used to investigate the cell invasion ability. Approximately 20 000 cells were seeded into each well (24‐well plates), which had been pre‐coated with 100 μl of diluted Matrigel with medium (1:6). Medium with 10% FBS was added to the lower chambers while serum‐free medium was added to the upper chambers. After incubation in 37°C for 24 or 48 h, the cells were stained with 0.5% crystal violet and observed under a microscope. A wound healing assay was performed to evaluate the migration ability of NSCLC cells.

### RNA pull‐down assay

RNA pull‐down assay was performed as described previously.^22^ RNA‐binding proteins (RBPs) associated with circRARS were identified using the maltose‐binding protein (MBP) affinity purification method. The MS2‐MBP protein was expressed in *Escherichia coli* and purified in accordance with a protocol from the Steitz laboratory. Three bacteriophage MS2 coat protein‐binding sites (5′‐CGTACACCATCAGGGTACGAGCTAGCCCATGGCGTACACCATCAGGGTACGACTAGTAGATCTCGTACACCATCAGGGTACG‐3′) were inserted downstream of circRARS by site‐directed mutagenesis with a Stratagene QuikChange Site‐Directed Mutagenesis Kit. To obtain RBPs associated with circRARS, A549 cells were transfected with 50 μg MS2‐tagged circRARS constructs, and 1 × 10^7^ cells were used for each RNA pull‐down assay.

### Glucose consumption, lactate production, and LDHA activity

Glucose consumption was assessed using a glucose assay kit (Solarbio) according to the manufacture's protocol. The cells were collected into centrifuge tubes and the supernatant was discarded after centrifugation. One milliliter of distilled water was added to 5 million cells. The cells were ultrasonicated (ice bath, power 200 W, ultrasound for 3 s, interval of 10 s, repeat 30 times) and placed in a boiling water bath for 10 min. After cooling, the samples were centrifuged for 10 min at 8000*g* and the supernatant was collected. The microplate reader was preheated for 30 min and the wavelength was adjusted to 505 nm. The microplates were then incubated at 37°C for 15 min in a water bath after adding the blank tube, standard tube, and test tube accroding to the manufacture's instruction. Finally, the glucose content was calculated according to the formulas provided in the instructions. Lactic acid production was evaluated using a lactate assay kit (Abbkine). Briefly, 5 × 10^6^ cells were collected in a centrifuge tube and washed with cold PBS. The supernatant was discarded after centrifugation. Next, 1 ml of lactate assay buffer was added to ultrasonically disrupt the cells for approximately 5 min (power 200 W, ultrasonic 3 s, interval 7 s, repeat 30 times). The samples were centrifuged at 12 000*g* for 5 min at 4°C. The supernatant was used for the assay and placed on ice to be tested. The microplate reader was preheated for more than 30 min and the wavelength was adjusted to 450 nm. Then, the microplates were incubated at 37°C for 30 min in the dark after adding the blank well, standard well, and test well. The absorbance value was measured at 450 nm. The blank well was recorded as *A*_Blank_, the standard well was marked *A*_Standard_, and the test well was marked *A*_Test_. Finally, Δ*A*_Test_ = *A*_Test_ − *A*_Blank_ and Δ*A*_Standard_ = *A*_Standard_ − *A*_Blank_ were calculated. A lactate dehydrogenase activity kit was used to investigate LDHA activity (Solarbio). The cells were collected in a centrifuge tube. The liquid in the upper layer was discarded after centrifugation. The cell amounts (10^4^):extract solution volume (ml) ratio was 500:1. The cells were split by ultrasonication (placed on ice, 200 W, work time 3 s, interval 10 s, repeated 30 times). The samples were centrifuged at 6900 *g* at 4°C for 10 min, and the supernatant was collected and placed on ice for testing. The microplate reader was preheated for 30 min and the wavelength was adjusted to 450 nm. For the sodium pyruvate standard solution, 100 μl of standard solution was diluted to 1, 0.5, 0.25, 0.125, and 0 μmol/ml, and 2, 1, 0.5, 0.25, 0.125, and 0 μmol/ml were used as a standard curve. Relevant reagents are added into three groups of test tubes, control tubes and standard tubes according to the instructions and mixed thoroughly. A total of 200 μl of reaction solution was placed in 96‐well flat‐bottom plates and the absorbance was measured at 450 nm after incubation at room temperature for 3 min. Finally, LDHA activity was calculated according to the formulas provided in the instructions.

### Statistical analysis

All quantitative data are presented as the means ± standard deviations of data from three independent experiments. All statistical analyses in this study were performed based on SPSS 22.0 software. Differences between two groups were analyzed by a paired two‐tailed *t*‐test. One‐way analysis of variance (ANOVA) or the nonparametric Kruskal–Wallis test was used to evaluate the associations between circRARS expression and other characteristics. Kaplan–Meier analysis was used to determine the prognostic value of circRARS expression and patients with OS were divided into two groups according to the median expression of circRARS in tumor tissues. Differences of *p < 0.05, **p < 0.01, **p < 0.001, and ****p < 0.001 were considered statistically significant.

## RESULTS

### Identification and characterization of circRARS in lung adenocarcinoma (LUAD)

**FIGURE 1:**
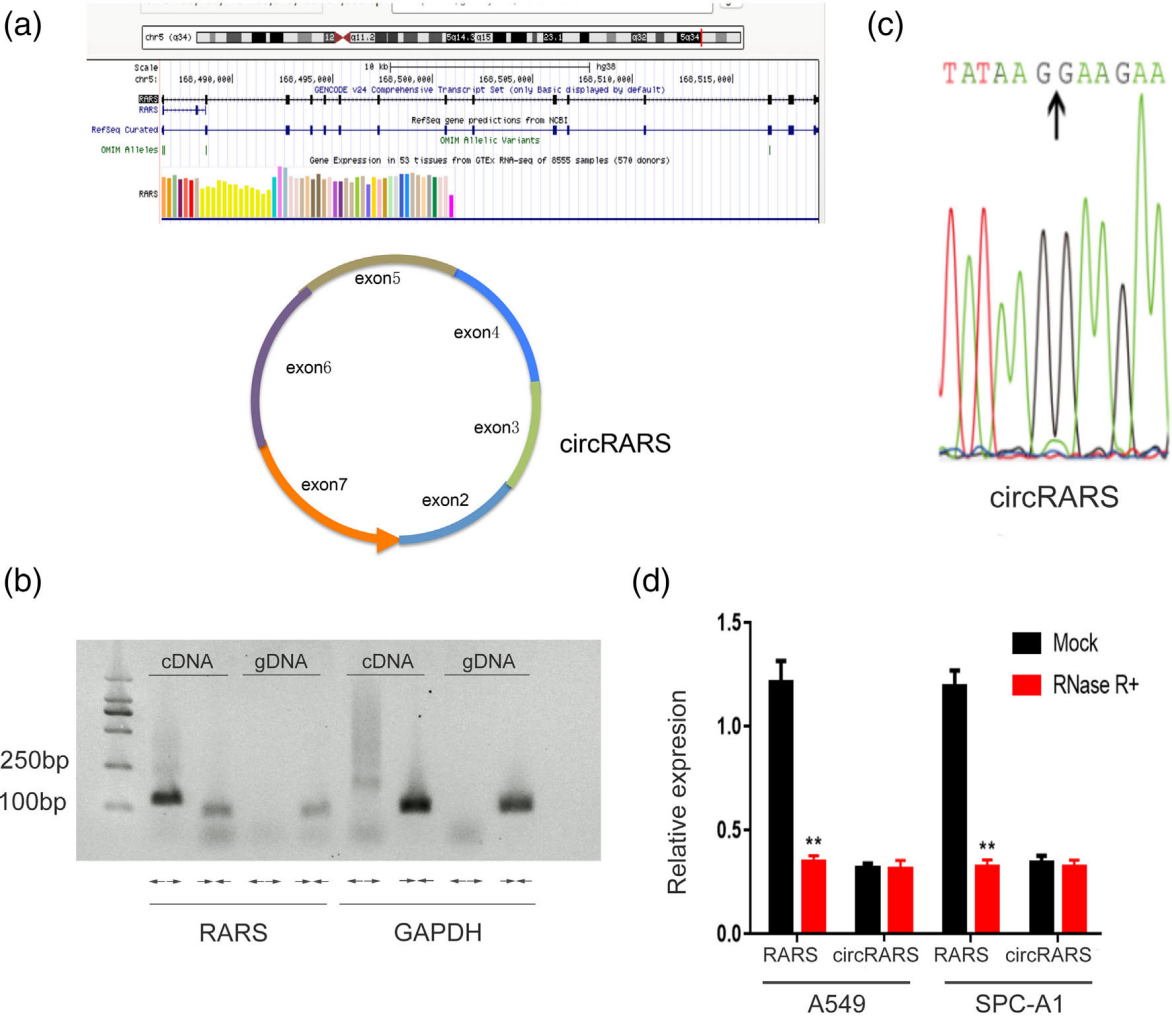
Circular RNA transcript of the RARS gene. (a) Location of circRARS and back‐splicing of exon2‐7. (b) Agarose gel electrophoresis analysis of PCR products of divergent primer and convergent primer. ← → divergent primers; → ← convergent primers. GAPDH was used as a control for a linear RNA transcript. cDNA, complementary DNA; gDNA, genomic DNA. (c) The splicing site of circRARS. (d) Relative expression level circRARS in A549 and SPC‐A1 with or without RNase +R digestion. **p < 0.01

Many differentially expressed circRNAs in LUAD were identified in our previous work.^23^ Using ribosomal RNA‐depleted RNA sequencing, we identified 3590 novel circRNA transcripts. A circular transcript of RARS (circBase ID: hsa_circ_0001551, named “circRARS” in this study) drew our interest. circRARS, a 777 nt circRNA transcript generated from back‐splicing of exons 2–7 of the RARS gene (Figure 1a), is highly overexpressed in LUAD tissues compared with the paired adjacent normal tissues.

We designed convergent primers and divergent primers to detect linear transcripts and circular transcripts, respectively. The convergent sequence was found to amplify only cDNA, which confirms the circular structure of the RNA transcript (Figure 1b). We also confirmed the back‐splicing site of circRARS by Sanger sequencing of PCR products (Figure 1c). To further confirm the circular characteristics of circRARS, we next digested RARS or circRARS with or without RNase R, which has exoribonuclease activity.^24^ RT‐PCR results showed that circRARS was resistant to RNase R compared with the linear transcripts in A549 and SPC‐A1 cells (Figure 1d).

### High level of circRARS is correlated with poor overall survival of NSCLC patients

**FIGURE 2:**
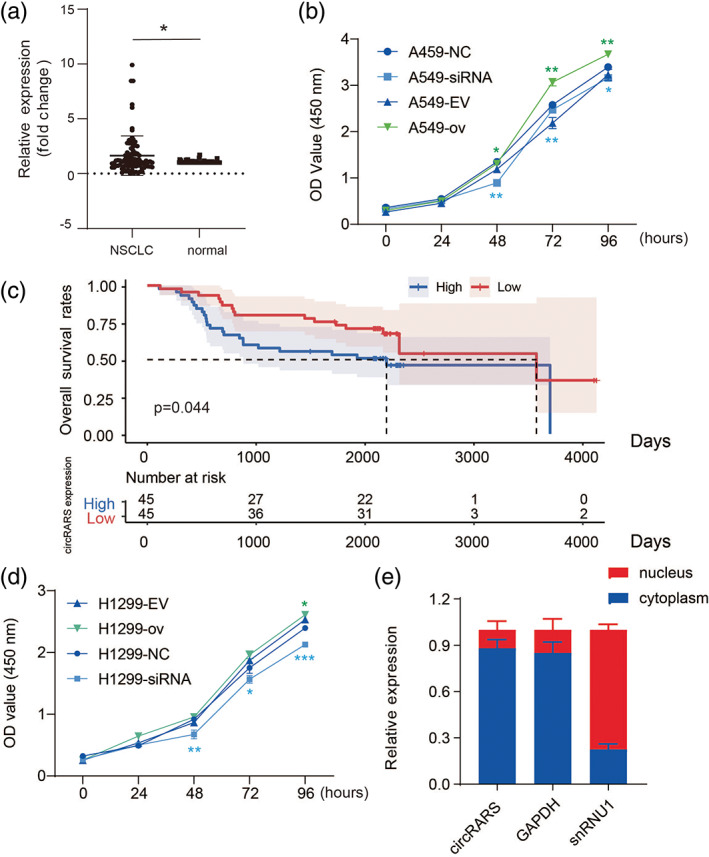
circRARS accelerated the proliferation of A549 and H1299 cells. (a) The circRARS expression in NSCLC tumor tissues and adjacent normal tissues. (c) The Kaplan–Meier curve showed higher expression of circRARS had worse overall survival rates. (b, d) CCK8 assays showed circRARS promoted the proliferation of A549 and H1299 cells. (*p < 0.05, **p < 0.01, ***p < 0.001). (e) circRARS was mainly located in the cytoplasm. NC, negative control; EV, empty vector; ov, circRARS overexpression

To determine the relationship between circRARS expression and clinical relevance, 90 primary NSCLC tumors and paired adjacent tissues were collected. The RT‐PCR results showed that circRARS was highly overexpressed in tumors compared with adjacent tissues (Figure 2a). Moreover, high expression of circRARS was significantly associated with smokers (*p* = 0.020), lymph node (LN) metastasis (*p* = 0.030), and higher tumor stage (*p* = 0.033) (Table S2). Kaplan–Meier analysis revealed that patients with high expression of circRARS had shorter overall survival times (*p* = 0.044) (Figure 2c), which suggests that upregulated expression of circRARS might be a prognostic biomarker in NSCLC. Univariable analysis illustrated that lymph node metastasis, advanced stage, and circRARS expression were significant indicators of OS in NSCLC patients (Table S3). However, only tumor stage, not circRARS expression, was an independent indicator of OS in NSCLC patients according to the multivariable analysis. In summary, NSCLC patients with high levels of circRARS had worse OS and upregulated expression of circRARS predicts the a worse prognosis.

### circRARS promoted NSCLC cells progression

We first analyzed the expression level of circRARS in different NSCLC cells and found that circRARS was more highly expressed in NSCLC cells than in 16‐HBE cells, a human bronchial epithelial cell line (Figure S1a). Next, we used siRNAs and plasmids to silence and overexpress circRARS, respectively (Figure S1b,c).

**FIGURE 3:**
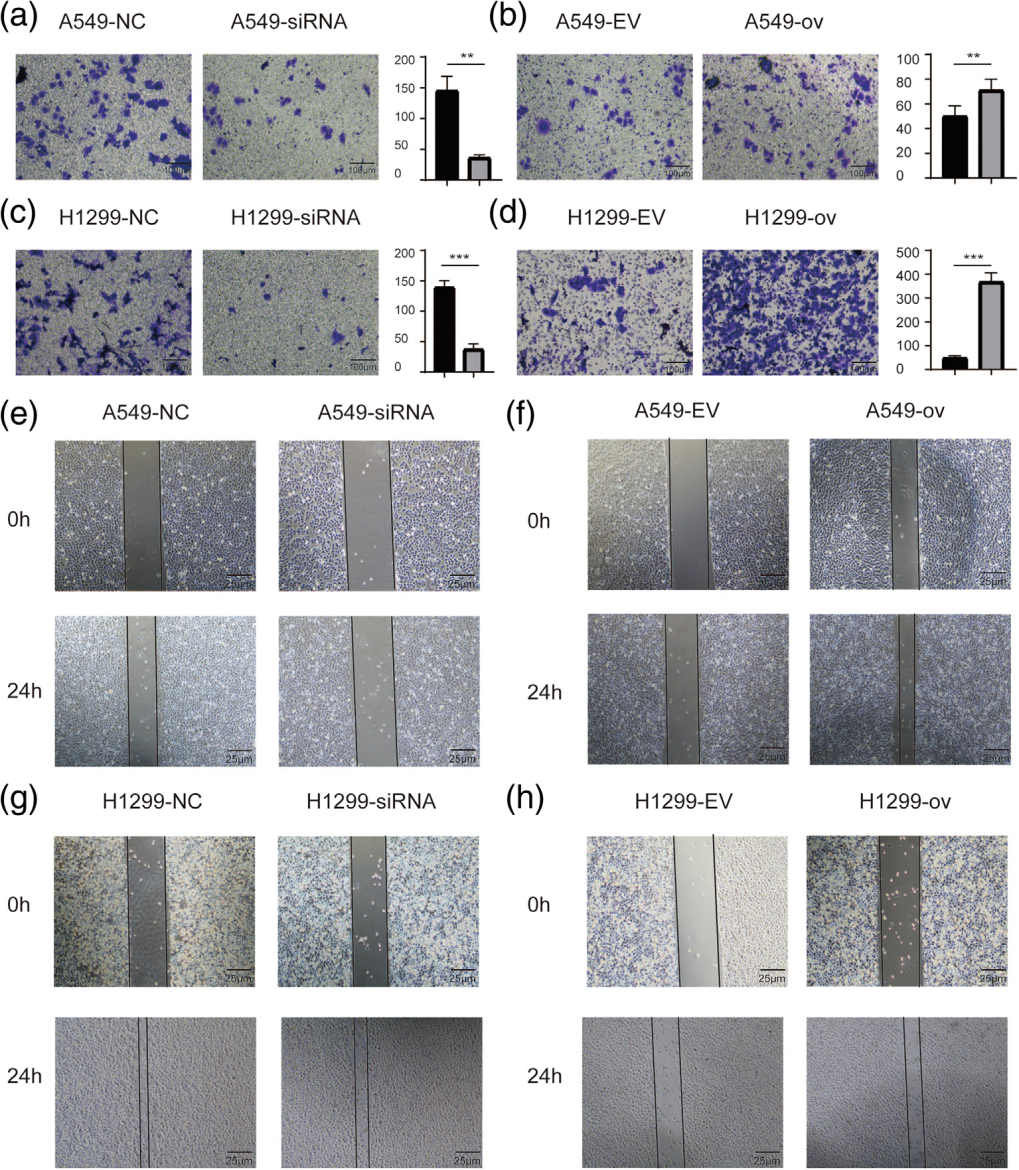
circRARS promoted the invasion and migration of A549 and H1299 cells. (a, c) Downregulated circRARS reduced the ability of invasion in A549 and H1299. (b, d) Upregulated circRARS accelerated the invasion in NSCLC cells. (e, g) Downregulated circRARS reduced the ability of migration in A549 and H1299. (f, h) Upregulated circRARS accelerated the migration in NSCLC cells. **p < 0.01, ***p < 0.001. NC, negative control; EV, empty vector; ov, circRARS overexpression

The cellular function of circRARS was further investigated in A549 and H1299 cells. CCK‐8 assays illustrated that silencing circRARS significantly decreased the proliferation ability of NSCLC cells but circRARS overexpression increased (Figure 2b,d). Transwell and Matrigel invasion assays illustrated that circRARS downregulation reduced the invasion ability of A549 and H1299 cells (Figure 3a,c), whereas circRARS overexpression accelerated the invasion of these cells (Figure 3b,d). Finally, wound healing assays showed that decreased circRARS expression compromised the ability of migration of NSCLC cells and vice versa (Figure 3e–h). Taken together, these data demonstrated that circRARS promoted the progression of NSCLC cells.

### circRARS positively regulated LDHA activity

To explore the underlying mechanisms by which circRARS promotes the progression of NSCLC, we first investigated the subcellular location of circRARS. The RT‐PCR results showed that circRARS mainly accumulated in the cytoplasm (Figure 2e). Thus, we sought to determine whether circRARS could regulate certain cytoplasmic proteins through direct interaction. MS2 RNA pull‐down assays were performed to identify proteins that can bind with circRARS. We constructed a vector expressing MS2‐labeled circRARS and a vector expressing only MS2. Compared with MS2 alone, mass spectrometry showed that 55 proteins were pulled down by MS2‐labeled circRARS only (Table S4). Among these proteins, LDHA drew our attention since LDHA is the final key enzyme in aerobic glycolysis.

**FIGURE 4:**
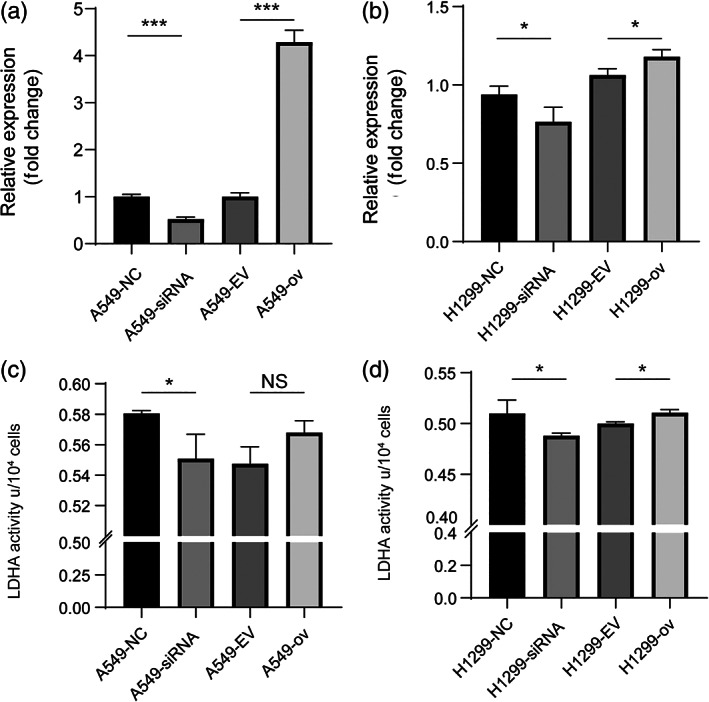
circRARS regulated LDHA expression at the transcriptional level and its enzyme activity. (a, b) circRARS positively regulated LDHA expression at the transcriptional level. (c, d) circRARS positively regulated LDHA activity. *p<0.05, ***p < 0.001

We then analyzed the relationship between circRARS and LDHA. First, we investigated LDHA expression at the mRNA level. At the transcriptional level, LDHA expression was significantly upregulated when circRARS was overexpressed and vice versa in A549 and H1299 cells (Figure 4a,b). Next, we wondered whether circRARS can affect LDHA reactivity, therefore we evaluated LDHA activity using the manufacturer's activity kit. The results showed that circRARS increased LDHA activity (Figure 4c,d). Taken together, we showed that circRARS can bind to LDHA and increase enzyme reactivity.

### circRARS accelerated aerobic glycolysis in NSCLC cells

**FIGURE 5:**
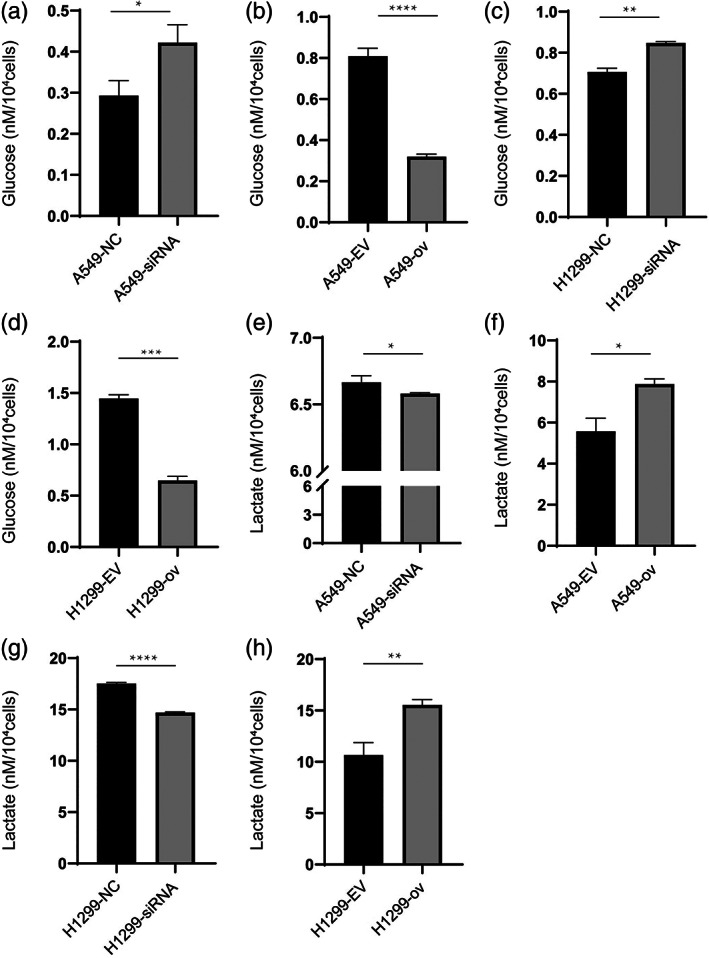
circRARS promoted glucose consumption and lactate production. (a, c) Knockdown circRARS decreased glucose consumption. (b, d) Overexpressed circRARS increased glucose consumption. (e, g) Downregulated circRARS reduced lactate production. (f, h) Upregulated circRARS promoted lactate production. *p < 0.05, **p < 0.01, ***p < 0.001, ****p < 0.0001. NC, negative control; EV, empty vector; ov, circRARS overexpression

As circRARS positively regulated the activity of LDHA, which is the rate‐limiting enzyme in the last step of glycolysis, we sought to answer the question of whether circRARS could subsequently affect the metabolic phenotype in NSCLC cells. The expression of circRARS was changed in A549 and H1299 cells using siRNAs and overexpression plasmids. Glucose consumption and lactate production were then detected in these cells. The results showed that downregulated circRARS decreased glucose consumption and lactate production (Figure 5a,c,e,g). However, this effect of circRARS on aerobic glycolysis can be reversed when circRARS are overexpressed (Figure 5b,d,f,h). Taken together, these results demonstrate that circRARS accelerates aerobic glycolysis in NSCLC cells.

### circRARS promoted NSCLC progression by regulating LDHA activity

**FIGURE 6:**
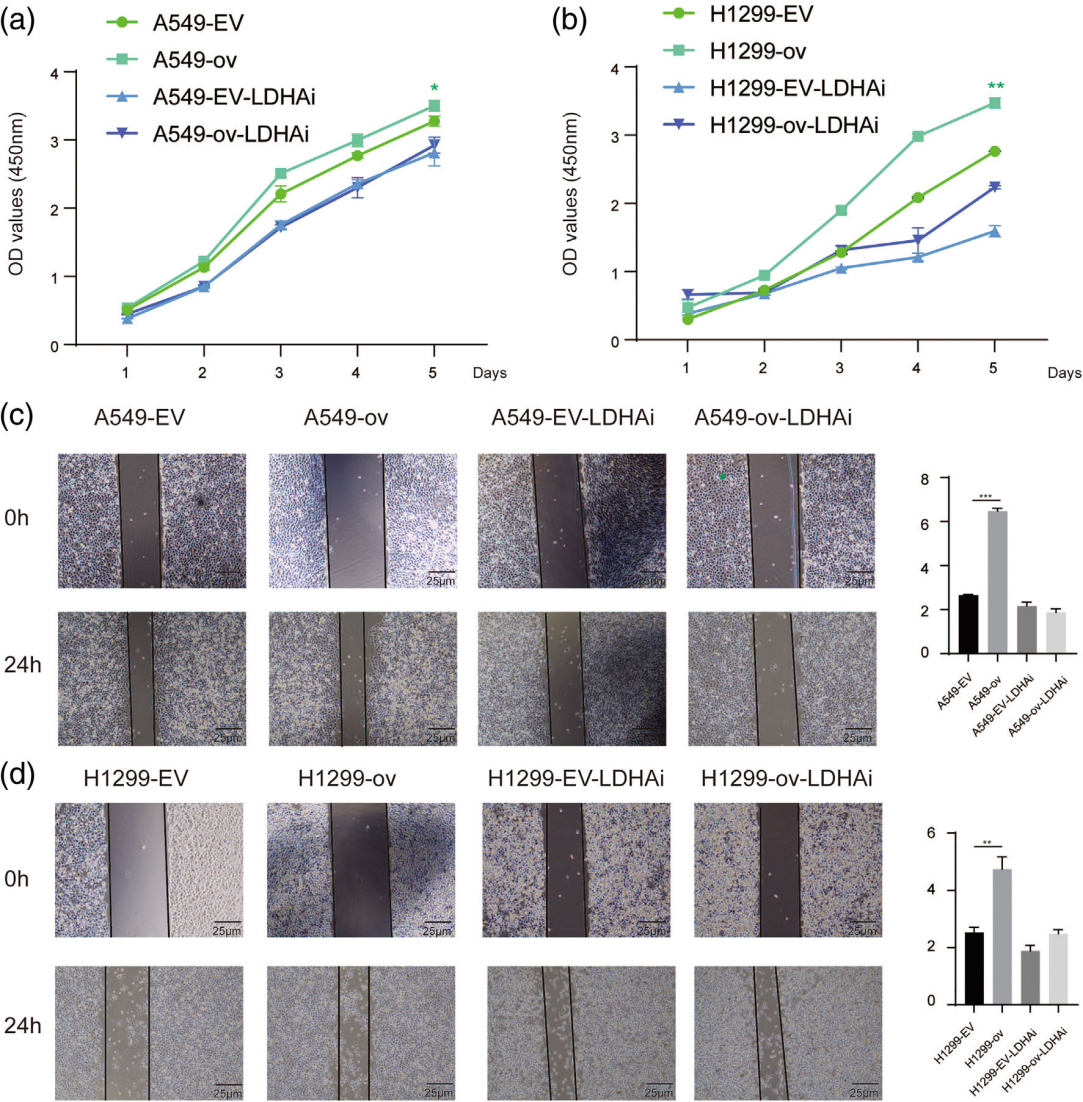
circRARS promoted NSCLC progression via regulating LDHA activity. Cell proliferation was determined by CCK8 assay in A549 (a) and H1299 (b). (c, d) Representative photos of the wound healing assays. After the indicated treatment, images of A549 and H1299 cells were taken at 0 and 24 h after scratching. *p < 0.05, **p < 0.01, ***p < 0.001. NC, negative control; EV, empty vector; ov, circRARS overexpression

To further examine whether circRARS affected NSCLC proliferation by regulating LDHA activity, we treated NSCLC cells with GSK2837808A (LDHAi, 20 μM), an effective selective inhibitor of LDHA activity. As shown by the CCK‐8 assay results (Figure 6a), overexpressed circRARS in A549 cells stimulated cancer cell proliferation, but the proliferative advantage was compromised by GSK2837808A. These results were also found in H1299 cells (Figure 6b). Wound healing assays showed that increased expression of circRARS promoted the migration of A549 and H1299 cells but LDHAi reduced this phenomenon (Figure 6c,d). This suggests that circRARS promotes NSCLC cell progression by regulating LDHA activity.

## DISCUSSION

Accumulating evidence has suggested that circRNAs perform vital roles in tumorigenesis, progression, invasion, and metastasis in LC.^8^, ^10^, ^25^ Recently, many studies have illustrated that circRNAs exert biological functions by acting as miRNA sponges or ceRNAs and translating peptides whose functions are mostly analogous to the full‐length protein counterparts.^26^ Another vital function is performed by circRNA‐protein interactions, especially RBPs like EIF4A3.^27^, ^28^ However, the underlying mechanisms of circRNA‐regulated proteins remains largely unknown.

In this study, we reported that circRARS was highly expressed in NSCLC and was closely associated with smoking status, LN metastasis, and tumor stage. Moreover, NSCLC patients with upregulated circRARS expression had worse OS. Functionally, knockdown circRARS in A549 and H1299 cells compromised cell proliferation, invasion, and migration, therefore circRARS could serve as an oncogene in NSCLC.

Many circRNAs are conserved in eukaryotes and can play different molecular functions because of distinct subcellular positions.^29^ Subcellular fraction assays showed circRARS mainly accumulated in the cytoplasm, indicating circRARS might directly regulate cytoplasmic proteins, therefore RNA pull‐down assay was performed and many proteins were pulled down. LDHA, the last catalytic enzyme of aerobic glycolysis, drew our interest. Seth et al. reported that inactivation of LDHA in oncogenic K‐RAS or EGFR NSCLC mouse models led to reduced tumorigenesis and disease regression and showed that LDHA is essential for cancer‐initiating cell survival and proliferation using an LDHA‐specific small molecule inhibitor.^30^ Our study demonstrated that circRARS can positively regulate LDHA activity and expression at the transcriptional level. However, whether circRARS regulated LDHA activity by shifting its dimer structure or inactivation or decreasing its degradation remains to be further investigated.

Functional assay in vitro demonstrated circRARS could accelerate aerobic glycolysis and serve as an oncogene. The abnormal glucose metabolic pathway in tumor cells is that glucose always converts glucose into lactate to produce adenosine triphosphate, which is called aerobic glycolysis or the Warburg effect.^31^ Previous studies have shown that circRNAs can promote LC tumorigenic and development by regulating aerobic glycolysis.^32^, ^33^ Wang and colleagues found circ‐MEMO1 knockdown decreased glucose uptake and lactate production in NSCLC cells, thus circ‐MEMO1 accelerated the glycolysis of NSCLC cells.^32^ Our study also revealed overexpressed circRARS could increase glucose consumption and lactate production. Taken together, it appears that circRNA can broadly regulate aerobic glycolysis to promote tumor development.

Several studies have reported that circRNAs can regulate LDHA expression or its activity to accelerate NSCLC progression.^21^, ^32^, ^34^, ^35^ Of note, hsa_circ_0002130 was highly expressed in Osimertinib‐resistant NSCLC cells and hsa_circ_0002130 can regulate LDHA through the ceRNA mechanism.^35^ It is therefore vital that circRNA regulates LDHA expression or activity because this mechanism not only influences the development of tumors but also the therapy of malignant carcinomas. In our study, we also demonstrated that circRNA can regulate LDHA activity and its expression at the transcriptional level to accelerate NSCLC progression. The data in our study indicate that circRARS might be a potentially effective therapeutic target for NSCLC.

In conclusion, we identified a novel circRNA, circRARS, which is overexpressed in NSCLC tissues and cells. Moreover, circRARS can promote NSCLC cell proliferation, invasion, and migration. circRARS can positively regulate LDHA activity and its expression at the transcription level and subsequently increase the progression of NSCLC cells. Our study showed that circRARS may serve as a biomarker for poor prognosis and a therapeutic target in NSCLC.

## AUTHOR CONTRIBUTIONS

All authors had full access to the data in the study and take responsibility for the integrity of the data and the accuracy of the data analysis. X.L. and M.Q.: conceptualization. H.L., Q.H., H.G., and X.C.: methodology. H.L. and Q.H.: formal analysis. H.L.: writing – original draft. X.L. and M.Q.: writing – review and editing. M.Q.: supervision. X.L., X.C. and M.Q.: funding acquisition.

## CONFLICT OF INTEREST

The authors declare no competing financial interests.

## Supporting information


**TABLE S1.** PCR primers and siRNA sequences

**TABLE S2.** Correlation between circRARS expression and clinicopathological characteristics in 90 NSCLCs

**TABLE S3.** Univariate and multivariate analysis of factors associated with OS

**TABLE S4.** Mass spectrometry results of MS2 pull down

**Figure S1.** (a) The relative expression of circRARS in different NSCLC cells and HBE cells. (b, c) The efficacy of siRNAs and overexpressed plasmid to knock down the expression of circRARS in A549

## Data Availability

The datasets generated and/or analyzed during the current study are available from the corresponding author on reasonable request.
