# Supplementary material for: Circular RNA, circular RARS, promotes aerobic glycolysis of non‐small‐cell lung cancer by binding with LDHA
Source: Thorac Cancer. 2023 Jan 11;14(4):389–98. doi: 10.1111/1759-7714.14758 (PMC9891865; doi:10.1111/1759-7714.14758)
Supplement: Supplementary file 1 — TABLE S1. PCR primers and siRNA sequences [file TCA-14-389-s003.docx]

TableS1. PCR primers and siRNA sequences

| Genes | Chain | Sequence (5’-3’) |
| --- | --- | --- |
| circRARS | Forward | GAGGTTCTTGTGCCAGGGAAA |
|  | Reverse | ACCCTCAATGGCGGGTATGT |
| LDHA | Forward | TTGGTCCAGCGTAACGTGAAC |
|  | Reverse | CCAGGATGTGTAGCCTTTGAG |
| GAPDH | Forward | CGCTCTCTGCTCCTCCTGTTC |
|  | Reverse | ATCCGTTGACTCCGACCTTCAC |
| si-circRARS | Sense (5’-3’) | UCUUUUAUAAGGAAGAAGATT |
|  | Antisense (5’-3’) | UCUUCUUCCUUAUAAAAGATT |
| Negative control | Sense (5’-3’) | UUCUCCGAACGUGUCACGUTT |
|  | Antisense (5’-3’) | ACGUGACACGUUCGGAGAATT |
