# Supplementary material for: Circular RNA, circular RARS, promotes aerobic glycolysis of non‐small‐cell lung cancer by binding with LDHA
Source: Thorac Cancer. 2023 Jan 11;14(4):389–98. doi: 10.1111/1759-7714.14758 (PMC9891865; doi:10.1111/1759-7714.14758)
Supplement: Supplementary file 2 — TABLE S2. Correlation between circRARS expression and clinicopathological characteristics in 90 NSCLCs [file TCA-14-389-s004.doc]

Table S2. Correlation between circRARS expression and clinicopathological characteristics in 90 NSCLCs.

| Variables | No. Patients | circRARS expression level | | |
| --- | --- | --- | --- | --- |
|  |  | **High** | **Low** | **P-value** |
| Age |  |  |  |  |
| <60 | 30 | 16 | 14 | 0.655 |
| ≥60 | 60 | 29 | 31 |  |
| Smoking state |  |  |  |  |
| smokers | 43 | 27 | 16 | **0.020** |
| Non-smokers | 47 | 18 | 29 |  |
| Gender |  |  |  |  |
| male | 55 | 31 | 24 | 0.130 |
| female | 35 | 14 | 21 |  |
| Histological type |  |  |  |  |
| Squamous cell  carcinoma | 31 | 19 | 12 | 0.120 |
| Adenocarcinoma | 59 | 26 | 33 |  |
| Lymph node metastasis |  |  |  |  |
| Yes | 34 | 22 | 12 | **0.030** |
| No | 56 | 23 | 33 |  |
| Tumor stage |  |  |  |  |
| I | 38 | 14 | 24 | **0.033** |
| II–IV | 52 | 31 | 21 |  |
| Tumor size |  |  |  |  |
| <3 cm | 41 | 18 | 23 | 0.290 |
| ≥3 cm | 49 | 27 | 22 |  |

**NOTE:** Bold values are statistically significant (*P*< 0.05).
