# Supplementary material for: Circular RNA, circular RARS, promotes aerobic glycolysis of non‐small‐cell lung cancer by binding with LDHA
Source: Thorac Cancer. 2023 Jan 11;14(4):389–98. doi: 10.1111/1759-7714.14758 (PMC9891865; doi:10.1111/1759-7714.14758)
Supplement: Supplementary file 3 — TABLE S3. Univariate and multivariate analysis of factors associated with OS [file TCA-14-389-s002.doc]

**Table S3:** Univariate and multivariate analysis of factors associated with OS.

| Variables | Univariate analysis | | |  | Multivariate analysis | | |
| --- | --- | --- | --- | --- | --- | --- | --- |
|  | HR | 95% CI | p |  | HR | 95% CI | p |
| Gender  (male *vs.* female) | 0.527 | 0.263-1.057 | 0.071 |  |  |  |  |
| Smoking status  (yes *vs.* no) | 1.858 | 0.989-3.529 | 0.054 |  |  |  |  |
| Tumor size  (≥3 cm *vs.*< 3 cm) | 1.045 | 0.558-1.958 | 0.890 |  |  |  |  |
| Lymph node metastasis  (yes *vs.* no) | 2.372 | 1.247-4.551 | **0.008** |  | 1.132 | 0.552-2.452 | 0.754 |
| Tumor stage  (II–IV *vs.* I) | 3.501 | 1.657-7.397 | **0.001** |  | 2.942 | 1.203-7.198 | **0.018** |
| circRARS level  (high *vs*. low) | 1.093 | 1.008-3.594 | **0.044** |  | 1.361 | 0.697-2.659 | 0.367 |

**Abbreviations and note:** OS, overall survival; 95% CI, 95% confidence interval; multivariate analysis, Cox proportional hazards regression model. Variables were adopted for their prognostic significance by univariate analysis with forward stepwise selection (forward, likelihood ratio). Variables were adopted for their prognostic significance by univariate analysis (p < 0.05).
