# Supplementary figures and images for: Circular RNA, circular RARS, promotes aerobic glycolysis of non‐small‐cell lung cancer by binding with LDHA
Source: Thorac Cancer. 2023 Jan 11;14(4):389–98. doi: 10.1111/1759-7714.14758 (PMC9891865; doi:10.1111/1759-7714.14758)

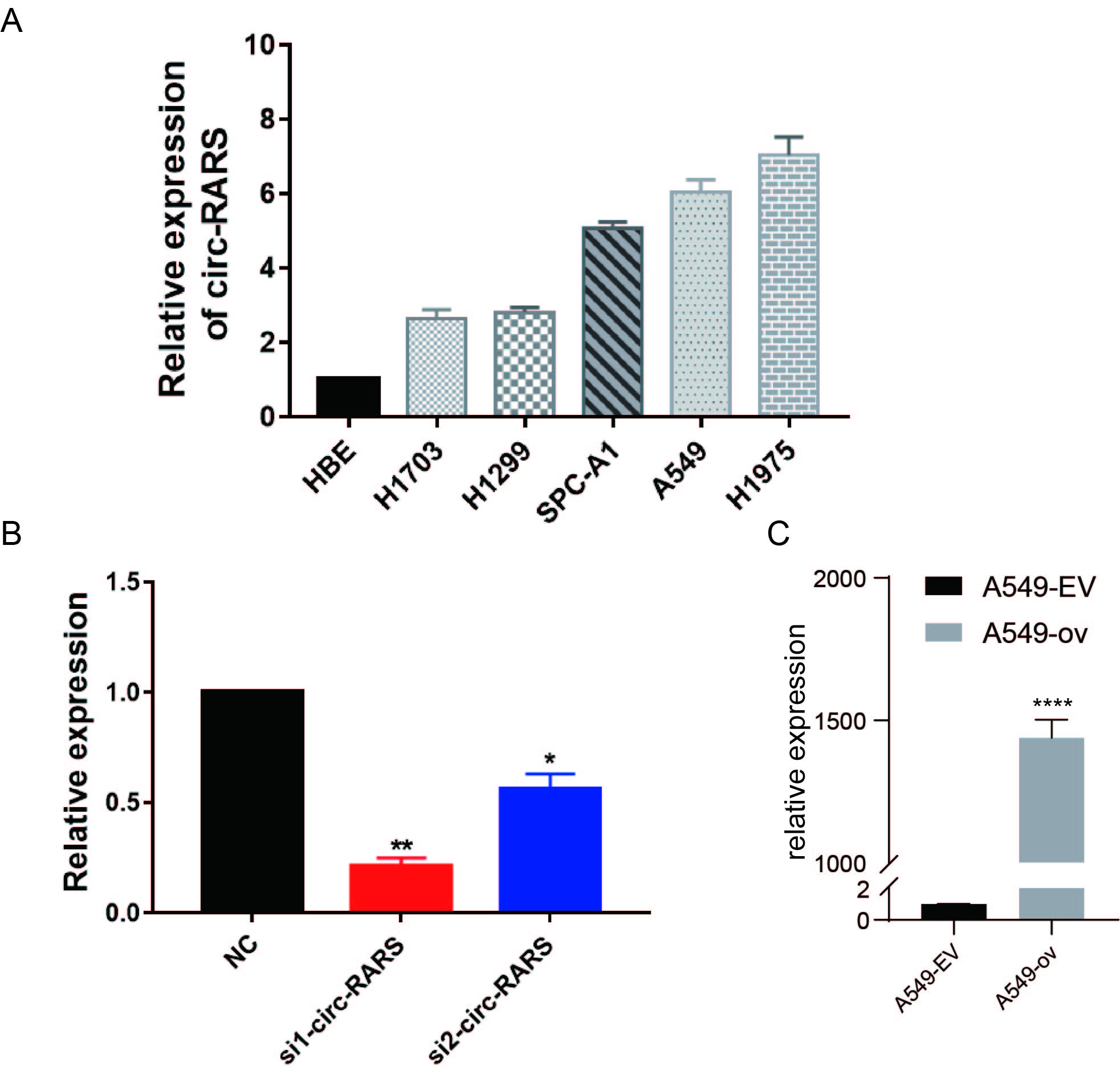

Supplement: Supplementary file 5 — Figure S1. (a) The relative expression of circRARS in different NSCLC cells and HBE cells. (b, c) The efficacy of siRNAs and overexpressed plasmid to knock down the expression of circRARS in A549 [file TCA-14-389-s005.jpg]
